# Time-varying discrimination accuracy of longitudinal biomarkers for the prediction of mortality compared to assessment at fixed time point in severe burns patients

**DOI:** 10.1186/s12873-020-00394-z

**Published:** 2021-01-06

**Authors:** Jaechul Yoon, Dohern Kym, Jun Hur, Jae Hee Won, Haejun Yim, Yong Suk Cho, Wook Chun

**Affiliations:** 1 Department of Surgery and Critical Care, Burn Center, Hangang Sacred Heart Hospital, College of Medicine, Hallym University Medical Center, 12, Beodeunaru-ro 7-gil, Youngdeungpo-gu, Seoul, 07247 South Korea; 2 Graduate School of Medicine, Kangwon National University, Chuncheon, Republic of Korea

**Keywords:** ROC, Time-varying, Discrimination, Mortality, Longitudinal, Burns

## Abstract

**Background:**

The progression of biomarkers over time is considered an indicator of disease progression and helps in the early detection of disease, thereby reducing disease-related mortality. Their ability to predict outcomes has been evaluated using conventional cross-sectional methods. This study investigated the prognostic performance of biomarkers over time.

**Methods:**

Patients aged > 18 years admitted to the burn intensive care unit within 24 h of a burn incident were enrolled. Information regarding longitudinal biomarkers, including white blood cells; platelet count; lactate, creatinine, and total bilirubin levels; and prothrombin time (PT), were retrieved from a clinical database. Time-dependent receiver operating characteristic curves using cumulative/dynamic and incident/dynamic (ID) approaches were used to evaluate prognostic performance.

**Results:**

Overall, 2259 patients were included and divided into survival and non-survival groups. By determining the area under the curve using the ID approach, platelets showed the highest c-index [0.930 (0.919–0.941)] across all time points. Conversely, the c-index of PT and creatinine levels were 0.862 (0.843–0.881) and 0.828 (0.809–0.848), respectively.

**Conclusions:**

Platelet count was the best prognostic marker, followed by PT. Total bilirubin and creatinine levels also showed good prognostic ability. Although lactate was a strong predictor, it showed relatively poor prognostic performance in burns patients.

**Supplementary Information:**

The online version contains supplementary material available at 10.1186/s12873-020-00394-z.

## Background

Burns is one of the most devastating traumas and results in high morbidity and mortality. Thus, predicting the adverse effects of interest or mortality using updated biomarkers that are measured routinely over time is an essential part of care in an intensive care unit. The progression of biomarkers over time is considered as an indicator of disease progression and is helpful for the early detection of disease [1]. Many biomarkers that are measured at a single time, such as at admission or due to a specific event [2], such as the development of acute kidney injury (AKI) or intervention, have been used to predict the outcomes at multiple time points of interest. The disease state of an individual changes over time; therefore, prognostic information, such as updated biomarkers recorded during routine measurements, also changes, possibly affecting the performance of decision-making tools.

The concept of the accuracy of sensitivity and specificity is fundamental to clinical research and decision modeling. Recently, statistical methods have been developed to generalize these traditional cross-sectional accuracy concepts for determining the time-varying characteristics of disease states [3]. In clinical practice, the ability of biomarkers to predict outcomes has been evaluated using conventional, cross-sectional methods [4]. Therefore, in this study, we investigated the prognostic potential of biomarkers routinely used in clinical practice over time and compared whether different biomarkers have varying prognostic accuracies at different times during treatment.

## Methods

From February 2007 to December 2018, patients aged > 18 years who were admitted at the burn intensive care unit (BICU) of Hangang Sacred Heart Hospital, Hallym University Medical Center within 24 h of the burn incident were included in this retrospective study; all the patients underwent acute fluid resuscitation during the first 3 days after the burn. The indications of admission to BICU were as follows: 1) partial or full thickness burn of > 20% of the total body surface area (TBSA) for adults and partial or full thickness burn of > 10% of the TBSA in patients aged > 65 years, 2) inhalation injury, 3) electrical burn, 4) preexisting medical disorder that could incur complications or affect mortality, and 5) concomitant trauma that could elevate the risk of morbidity or mortality. Inhalation injuries were diagnosed by a combination of history (burned in an enclosed space, unconscious at scene, prolonged extrication), physical findings such as singed facial hair, carbonaceous deposits in the nose or mouth, and facial burns. Clinical longitudinal data that were measured routinely and were known predictors, such as white blood cell (WBC) count, blood platelet count, serum lactate concentrations [5], serum creatinine concentrations, serum total bilirubin (TB) concentrations, and blood prothrombin time (PT), were retrieved from a clinical database warehouse at Hangang Sacred Heart Hospital. Serum myoglobin, serum lactate dehydrogenase, and blood pH, which were used in Hangang, were excluded because they were routine tests at admission, were not measured longitudinally, and blood pH was associated with lactate. The study period was the stay in the BICU. When the biomarkers were measured several times each day, the poorest daily value was recorded. Demographic variables, such as age, sex, TBSA (calculated by a surgeon using a modified Lund and Browder chart) [6], type of burn, length of BICU stay, and presence of inhalation injury [7], were also noted. The primary outcome was death in the BICU. The severity of injury was reported using the Abbreviated Burn Severity Index (ABSI) [8], which is a newly developed Hangang score [9] at our center, and the Acute Physiology and Chronic Health Evaluation Score (APACHE) IV [10].

### Burn management

All patients admitted to the BICU received initial fluid resuscitation using the modified Parkland formula (4 mL × kg × %TBSA burned) and the amount of fluid volume was adjusted to maintain a minimum urine output of 0.5 ml/kg/h. Enteral feeding was the first choice and began within 48 h if there was no ileus, and this was supplemented with parenteral nutrition to meet caloric goals as measured by ESPEN guidelines for intensive care [11]. Burn wound dressing was performed daily with hydrofoam and topical antimicrobials. Early excision and grafting with autograft/allograft was performed within 5 days after admission.

### Statistical analyses

Baseline demographic characteristics were reported as follows. Continuous variables with normal distribution are presented as mean ± standard deviation (SD) values and variables with non-normal distribution are presented as medians (25th interquartile range [IQR]–75th IQR). Depending on data normality, the independent test or Wilcoxon signed-rank test was used to determine differences between the two groups. Categorical variables were analyzed using the Chi-square test and are presented as percentages. We used two methods of time-dependent receiver operating characteristic (ROC) curves to evaluate the prognostic performance using fixed baseline biomarkers measured at admission and updated the biomarkers measured routinely during the study period. We calculated the incident/dynamic (ID) ROC using a non-parametric rank-based approach and allocated subjects with an event at time into the positive group and those who experienced an event thereafter into the negative group [12]. The cumulative/dynamic (CD) ROC curve was developed by allocating subjects who experienced the event before the fixed point (at the end of each week) of time into the positive group and eventless subject during time into the negative group [13]. The data for CD ROC were subsetted to analyze the diagnostic performance every week from week 1 to 8. The difference in CD and ID approaches has been elucidated by Kamarudin et al. [2]. Confidence intervals (CIs) were calculated using 500-time bootstrap resampling, and percentile-based confidence intervals were obtained. Two side *p*-value < 0.05 was considered statistically significant. All analyses were conducted by using computing statistical R-project program version 3.6.

## Results

### Baseline characteristics of the survivors and non-survivors

In total, 2259 patients were included in this retrospective study; among them, 1786 patients were allocated to the survival group and 473 to the non-survival group; the overall mortality was 20.9%. The overall median age was 48.0 years and was higher in the non-survival group than in the survival group (52.0 vs. 46.0 years). The overall median burn TBSA was 24.0% and was significantly higher in the non-survival group (65.0%). Inhalation was significantly higher in the non-survival group (78.2%). The median APACHE IV, Hangang, and ABSI were 29, 39, and 5 points higher, respectively, in the non-survival group. All baseline laboratory results included in this study were collected at admission and were significantly different between the two groups (Table 1).

**Table 1:** Baseline characteristics between the two groups

Variables	Survivors(*n* = 1786)	Non-survivors(*n* = 473)	Total(*n* = 2259)	*p*-value
Age	46.0 [37.0;55.0]	52.0 [43.0;65.0]	48.0 [38.0;56.5]	< 0.001
Sex				0.495
Male	1469 (82.3%)	382 (80.8%)	1851 (81.9%)	
TBSA	24.0 [14.0;37.0]	65.0 [42.0;85.0]	29.0 [17.0;48.0]	< 0.001
Type				< 0.001
FB	1228 (68.8%)	421 (89.0%)	1649 (73.0%)	
EB	318 (17.8%)	9 (1.9%)	327 (14.5%)	
SB	156 (8.7%)	30 (6.3%)	186 (8.2%)	
CoB	50 (2.8%)	10 (2.1%)	60 (2.7%)	
ChB	34 (1.9%)	3 (0.6%)	37 (1.6%)	
Inhalation	812 (45.5%)	370 (78.2%)	1182 (52.3%)	< 0.001
APACHE IV	33.0 [24.0;45.0]	62.0 [49.0;76.0]	38.0 [26.0;53.0]	< 0.001
Hangang	122.0 [113.0;132.0]	161.0 [149.0;177.0]	127.0 [115.0;144.0]	< 0.001
ABSI	7.0 [6.0; 9.0]	12.0 [10.0;14.0]	8.0 [6.0;10.0]	< 0.001
LOS	15.0 [6.0;35.0]	12.0 [7.0;22.0]	14.0 [6.0;32.0]	0.001
Laboratory				
WBC(× 10^3^/uL)	17.3 [13.0;22.7]	29.2 [20.6;37.4]	18.7 [13.7;26.0]	< 0.001
Platelet(× 10^3^/uL)	230.5 [186.0;280.0]	193.0 [134.5;269.0]	225.0 [175.0;279.0]	< 0.001
Creatinie (mg/dL)	0.8 [0.6; 0.9]	1.0 [0.8; 1.4]	0.8 [0.7; 1.0]	< 0.001
Lactate (mmol/L)	2.6 [1.7; 4.0]	5.6 [3.9; 7.9]	3.0 [1.9; 5.0]	< 0.001
TB (mg/dL)	0.8 [0.5; 1.1]	1.1 [0.8; 1.7]	0.8 [0.6; 1.2]	< 0.001
PT (sec)	11.8 [10.9;12.9]	13.1 [11.8;14.9]	12.0 [11.0;13.3]	< 0.001

*n* number, *FB* Flame Burn, *SB* Scald Burn, *EB* Electrical Burn, *ChB* Chemical Burn, *CoB* Contact Burn, *%TBSA burned* percentage of total body surface area burned, *APACHE* Acute Physiology and Chronic Health Evaluation Score, *ABSI* Abbreviated Burn Severity Index, *LOS* length of hospital stay, *TB* total bilirubin, *PT* prothrombin time, *WBC* white blood cell

### Diagnostic performance of baseline and updated biomarkers over time using the ID approach

**Fig. 1:**
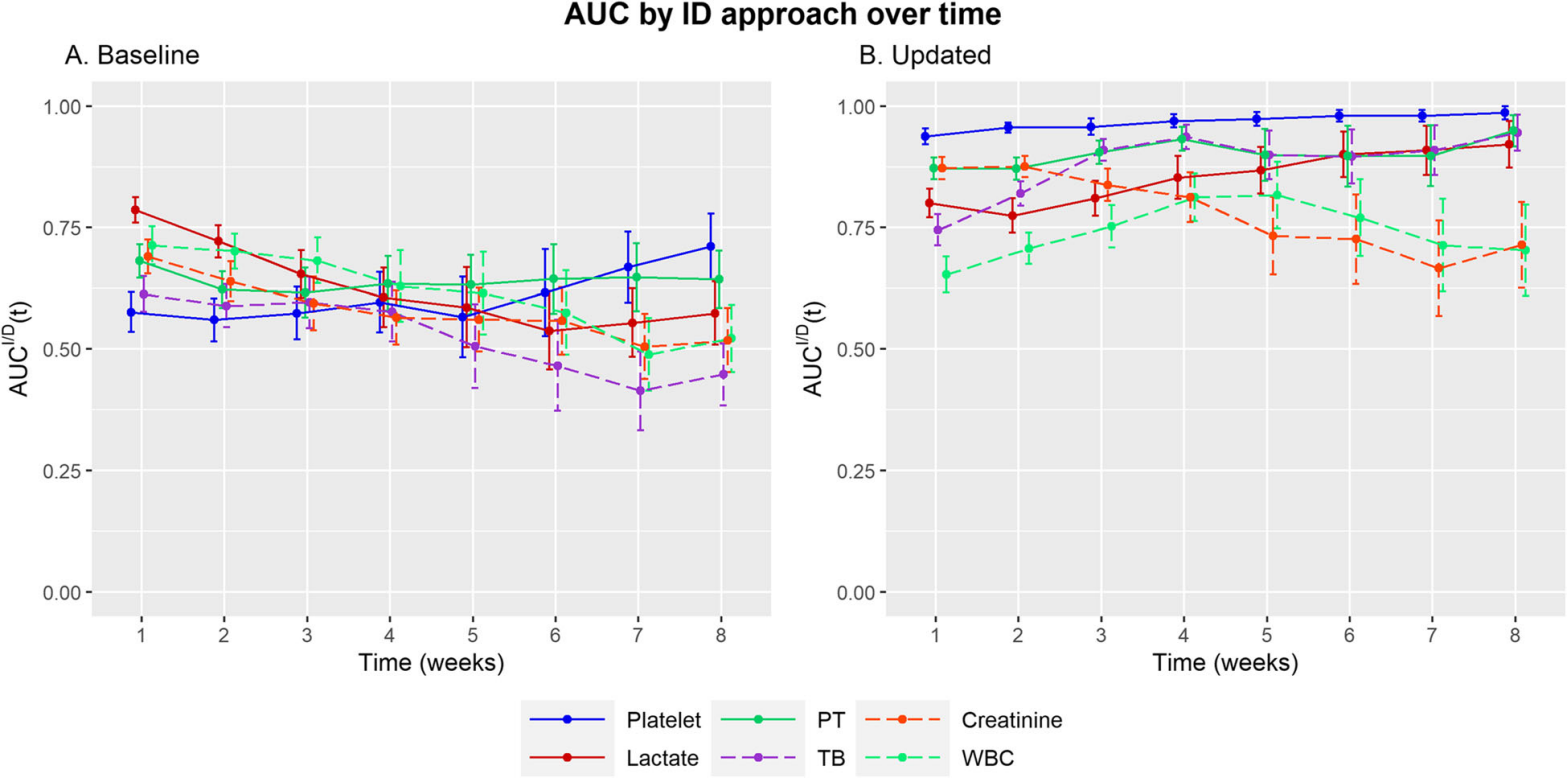
Diagnostic performance of the baseline and updated biomarkers over time using the ID approach. Error bar is 95%d confidence interval calculated using the bootstrap method

ID ROC curve are particularly well suited for assessing the performance of markers measured at a series of time points during decision-making [4]. First, the area under the curve (AUC) using baseline biomarkers (Additional file 1), and the c-index, which shows the overall performance of different biomarkers, was highest for lactate [0.662 (95% CI, 0.614–0.673)]. However, the AUC value of lactate ranged from 0.786 (0.760 ~ 0.812) in the 1st week to 0.574 (0.509–0.639) in the 8th week, showing a decreasing trend. The AUC value for platelet count, which had a lower c-index [0.576 (0.546–0.605)], ranged from 0.576 (0.535–0.617) in the 1st week to 0.711 (0.643–0.779) in the 8th week, and thus showed an increasing trend. For the updated biomarkers (Additional file 2), platelet count had the highest c-index [0.930 (0.919–0.941)] across all time points. PT and creatinine concentration showed over 8 c-index [0.862 (0.843–0.881) and 0.828 (0.809–0.848), respectively] (Fig. 1).

### Diagnostic performance of baseline and updated biomarkers over time using the CD approach

**Fig. 2:**
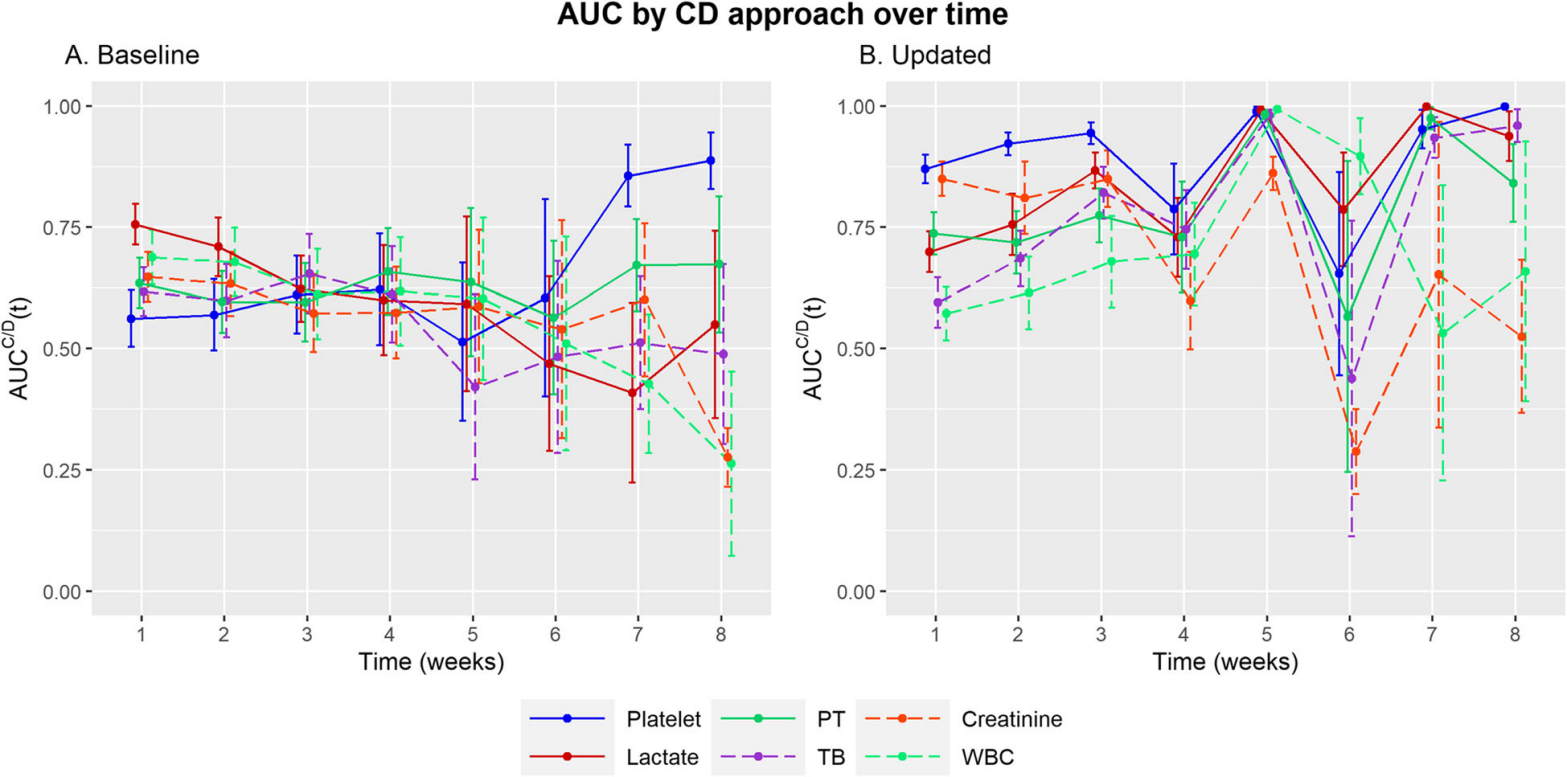
Diagnostic performance of the baseline and updated biomarkers over time by the CD approach. Error bar is 95%d confidence interval calculated using the bootstrap method

**Fig. 3:**
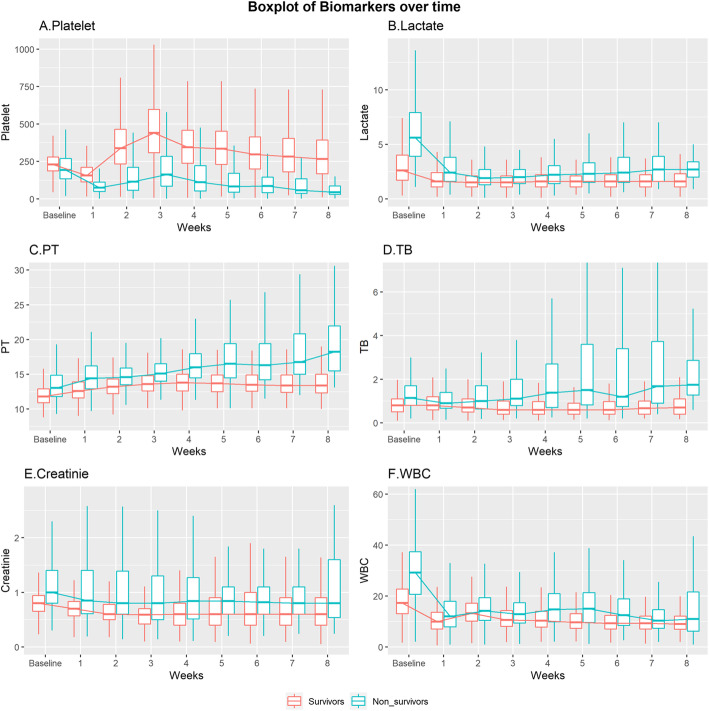
The boxplots of all biomarkers over 8 weeks

CD ROC curves are suitable tools for assessing prognostic accuracy when identifying individuals at risk of event before the time of interest [4]. First, in terms of AUC using baseline biomarkers (Additional file 3), the AUC values for platelet count ranged from 0.562 (0.504–0.62) in the 1st week to 0.888 (0.829–0.946) in the 8th week, showing an increasing trend. Lactate ranged from 0.756 (0.714–0.798) in the 1st week to 0.550 (0.356–0.743) in the 8th week, showing a decreasing trend. For the updated biomarkers (Additional file 4), platelet count showed the highest AUC value of 0.871 (0.841–0.900) in the 1st week and 0.999 (0.997–1.000) in the 8th week. Lactate showed the highest AUC value of 0.999 (0.998–1.000) in the 7th week, whereas PT showed a value of over 7, except at week 6 (0.566, 95% CI 245–0.887) (Fig. 2). The boxplots of all biomarkers over 8 weeks are shown in Fig. 3, and the number of patients calculated for each of the 8 weeks is presented in Table 2.

**Table 2:** The number of patients calculated for each of the eight weeks

	Week 1	Week 2	Week 3	Week 4	Week 5	Week 6	Week 7	Week 8
Non-Survivors	126	158	69	49	30	15	12	14
Survivors	2133	1384	1014	781	591	449	322	235

## Discussions

In the present study, we evaluated the time-varying diagnostic performance of biomarkers measured at admission and updated the biomarkers at several time points in routine clinical settings. If accurate predictions are made, they could suggest clinical recommendations for the selection and timing of interventions and help initiate specific preventive strategies and aggressive treatment for high-risk individuals. These approaches could reduce costs, adverse effects, and unnecessary interventions in low-risk patients. Ultimately, the goal of the prognostic model using biomarkers is to accurately predict the time of the event or to distinguish cases and controls in a variety of situations.

The performance of the updated biomarkers by the ID approach was higher than that of the baseline biomarkers in all situations, with the exception of the 1st week (0.713 vs. 0.653) for WBC (Additional files 1 and 2). The performance of the updated biomarkers by the CD approach was higher than that of the baseline biomarker in most parameters, except for lactate in the 1st week (0.756 vs. 0.699), WBC in the 1st (0.689 vs. 0.572) and 2nd (0.679 vs. 0.615) week, TB in the 1st (0.617 vs. 0.595) and 6th (0.483 vs. 0.438) week, and creatinine in the 6th week (0.540 vs. 0.288) (Additional files 3 and 4). From the results, we identified that patient biomarkers must be regularly updated to maintain prognostic accuracy because good prognostic markers effectively suggest the choice and timing of therapeutic interventions, allowing timely action for individuals with the greatest risk of complications.

The updated platelet biomarker had the highest c-index of 0.930 (95% CI, 0.919–0.941), which maintained the AUC ID over 0.930 over time, indicating that it is a strong prognostic biomarker for practical use. Moreover, we used AUC CD over a period of 1 week to actually evaluate the use of updated biomarkers as a decision tool. We found that the AUC CD of platelet was consistently higher than 0.870 across all selected time points except at weeks 4 and 6. This indicates that platelet count identifies high-risk patients at a high-risk of mortality. Cate et al. [14] reported that platelet count is a strong predictor of mortality and reported an AUC of 0.779 (95% CI 0.697–0.862), which was calculated by the value measured on the third day after admission. Huang et al. [15] reported that platelets could be a biomarker of mortality, with an AUC value of 0.782. However, baseline platelet count showed a lower AUC than other biomarkers during earlier times. Lactate has been used as a predictor of cellular hypoxia and shock, with an AUC value of 0.82, indicating high prognostic performance [16]. Adding lactate to the severity scores predicts mortality better in critically ill patients [17]. In our study, lactate showed a relatively lower c-index (0.786) than platelets, PT, and creatinine. This could be because lactate further reflects the severity of burn than that of mortality. Creatinine is also a better risk factor of AKI rather than that of mortality [18]. However, creatinine showed high discrimination, with a c-index of 0.828. This is probably because AKI is one of the most common complications in burn patients. PT also showed high discrimination, with a c-index of 0.862, and has been reported to be a predictor in many diseases, such as liver disease, cardiac disease, and trauma [19–21]. PT is reported as an early predictor of hepatic dysfunctions [22]. However, it was a good predictor throughout the study period.

Many baseline biomarkers have been suggested and used to predict the outcome in critically ill and burn patients at certain time points, such as at the time of admission. These biomarkers provide the status of the patients at a specific time. However, longitudinal biomarkers can provide more useful and varied information because repeated measurements of biomarkers over time offer physicians insights into the individual and the overall trajectory. The distribution of biomarkers over time could signal disease initiation and aid in the earlier detection of disease and in reducing mortality from disease. Therefore, although baseline biomarkers at specific time points can accurately predict the outcome, physicians also need clues as to the patient’s status using longitudinally updated biomarkers to make accurate prognoses.

This study has some limitations. First, it was not multicenter study; thus, our population does not represent the entire population of Korea. However, our center is the only unit run by the University of Korea. Second, we set an arbitrary window period of 1 week for the CD approach to compare the biomarkers; thus, we cannot conclude how often the biomarkers should be updated. The results should be interpreted with caution because the subgroups during the study period may include patients with different physiological conditions, and the causes of death may also be different. The causes of death in the early stages are usually associated with burn shock, acute respiratory failure, and AKI induced by the burn itself. The later causes are associated with complications as a result of burn care and include AKI and acute respiratory failure induced by sepsis. Therefore, the prognostic performance of the biomarkers reflecting these changes might vary over time, and the ID or CD approach at a specific time of interest may be more appropriate.

## Conclusions

For accurate predictions, biomarkers should be updated regularly. Platelet count showed the best prognostic performance, followed by PT. Creatinine concentration and TB could be prognostic factors for certain diseases, and their prognostic performance was good at specific time points. The overall prognostic performance of the biomarkers was good in burn patients; however, the pattern of creatinine concentrations, which were weak later, and of TB concentrations, which were weak earlier, is opposite. Lactate is known as a strong predictor, but it showed a relatively low prognostic performance in burn patients.

## Supplementary Information

**Additional file 1: Supplementary Table 1.** Time varying Performance of baseline biomarkers using ID approach (AUC with 95% CI).

**Additional file 2: Supplementary Table 2.** Time varying Performance of updated biomarker using ID approach (AUC with 95% CI).

**Additional file 3: Supplementary Table 3.** Time varying Performance of baseline biomarkers using CD approach (AUC with 95% CI).

**Additional file 4: Supplementary Table 4.** Time varying Performance of updated biomarker using CD approach (AUC with 95% CI).

## Data Availability

The datasets used and/or analyzed during the current study are available from the corresponding author upon reasonable request.

## Abbreviations

WBC: White blood cell
TB: Total bilirubin
PT: Prothrombin time
ROC: Receiver operating characteristic
AUC: Area under the curve
ID: Incident/dynamic
AKI: Acute kidney injury
BICU: Burn intensive care unit
TBSA: Total body surface area
ABSI: Abbreviated Burn Severity Index
APACHE: Acute Physiology and Chronic Health Evaluation Score
SD: Standard deviation
IQR: Interquartile range
n: Number
FB: Flame Burn
SB: Scald Burn
EB: Electrical Burn
ChB: Chemical Burn
CoB: Contact Burn
CD: Cumulative/dynamic

## References

[1] Han Y, Albert PS, Berg CD, Wentzensen N, Katki HA, Liu D (2020). Statistical approaches using longitudinal biomarkers for disease early detection: A comparison of methodologies. Statistics in medicine..

[2] Kamarudin AN, Cox T, Kolamunnage-Dona R (2017). Time-dependent ROC curve analysis in medical research: current methods and applications. BMC Med Res Methodol.

[3] Bansal A, Heagerty PJ (2018). A tutorial on evaluating the time-varying discrimination accuracy of survival models used in dynamic decision making. Med Decision Mak.

[4] Bansal A, Heagerty PJ (2019). A comparison of landmark methods and time-dependent ROC methods to evaluate the time-varying performance of prognostic markers for survival outcomes. Diagn Progn Res.

[5] Zhi L, Hu X, Xu J, Yu C, Shao H, Pan X, Hu H, Han C (2015). The characteristics and correlation between the ischemia-reperfusion and changes of redox status in the early stage of severe burns. Am J Emerg Med.

[6] Wachtel TL, Berry CC, Wachtel EE, Frank HA (2000). The inter-rater reliability of estimating the size of burns from various burn area chart drawings. Burns.

[7] Walker PF, Buehner MF, Wood LA, Boyer NL, Driscoll IR, Lundy JB, Cancio LC, Chung KK (2015). Diagnosis and management of inhalation injury: an updated review. Crit Care.

[8] Tobiasen J, Hiebert JM, Edlich RF (1982). The abbreviated burn severity index. Ann Emerg Med.

[9] Kim Y, Kym D, Hur J, Jeon J, Yoon J, Yim H, Cho YS, Chun W (2019). Development of a risk prediction model (Hangang) and comparison with clinical severity scores in burn patients. PLoS One.

[10] Zimmerman JE, Kramer AA, McNair DS, Malila FM (2006). Acute physiology and chronic health evaluation (APACHE) IV: hospital mortality assessment for today's critically ill patients. Crit Care Med.

[11] Kreymann KG, Berger MM, Deutz NE, Hiesmayr M, Jolliet P, Kazandjiev G, Nitenberg G, van den Berghe G, Wernerman J, Ebner C (2006). ESPEN guidelines on enteral nutrition: intensive care. Clin Nutr.

[12] Díaz-Coto S, Martínez-Camblor P, Pérez-Fernández S (2020). SmoothROCtime: an R package for time-dependent ROC curve estimation. Comput Stat..

[13] Zheng Y, Heagerty PJ (2007). Prospective accuracy for longitudinal markers. Biometrics.

[14] Cato LD, Wearn CM, Bishop JRB, Stone MJ, Harrison P, Moiemen N (2018). Platelet count: a predictor of sepsis and mortality in severe burns. Burns.

[15] Huang X, Guo F, Zhou Z, Chang M, Wang F, Dou Y, Wang Z, Huan J (2019). Relation between dynamic changes of platelet counts and 30-day mortality in severely burned patients. Platelets.

[16] Mokline A, Abdenneji A, Rahmani I, Gharsallah L, Tlaili S, Harzallah I, Gasri B, Hamouda R, Messadi A (2017). Lactate: prognostic biomarker in severely burned patients. Ann burns fire disasters.

[17] Aksu A, Gulen M, Avci A, Satar S (2019). Adding lactate to SOFA and qSOFA scores predicts in-hospital mortality better in older patients in critical care. Eur Geriatric Med.

[18] Kim Y, Cho YS, Kym D, Yoon J, Yim H, Hur J, Chun W (2018). Diagnostic performance of plasma and urine neutrophil gelatinase-associated lipocalin, cystatin C, and creatinine for acute kidney injury in burn patients: a prospective cohort study. PLoS One.

[19] Karaca M, Bayata MS, Nazlı C (2018). Prognostic value of Prothrombin time in patients with acute coronary syndrome undergoing percutaneous coronary intervention.

[20] Umebachi R, Taira T, Wakai S, Aoki H, Otsuka H, Nakagawa Y, Inokuchi S (2018). Measurement of blood lactate, D-dimer, and activated prothrombin time improves prediction of in-hospital mortality in adults blunt trauma. Am J Emerg Med.

[21] Gao F, Cai M-X, Lin M-T, Xie W, Zhang L-Z, Ruan Q-Z, Huang Z-M (2019). Prognostic value of international normalized ratio to albumin ratio among critically ill patients with cirrhosis. Eur J Gastroenterol Hepatol.

[22] Saleh SM (2018). The effect of moderate and severe burn injuries on human liver, kidney & blood (biochemical study). Zagazig J Forensic Med.

