# Supplementary material for: Time-varying discrimination accuracy of longitudinal biomarkers for the prediction of mortality compared to assessment at fixed time point in severe burns patients
Source: BMC Emerg Med. 2021 Jan 6;21:1. doi: 10.1186/s12873-020-00394-z (PMC7786914; doi:10.1186/s12873-020-00394-z)
Supplement: Supplementary file 1 — Additional file 1: Supplementary Table 1. Time varying Performance of baseline biomarkers using ID approach (AUC with 95% CI). [file 12873_2020_394_MOESM1_ESM.docx]

Supplementary Table 1. Time varying Performance of baseline biomarkers using ID approach (AUC with 95% CI)

|  | week1 | week2 | week3 | week4 | week5 | week6 | week7 | week8 | c-index |
| --- | --- | --- | --- | --- | --- | --- | --- | --- | --- |
| Platelet | 0.576 (0.535~0.617) | 0.560 (0.516~0.604) | 0.574 (0.52~0.628) | 0.597 (0.534~0.659) | 0.566 (0.483~0.649) | 0.616 (0.527~0.705) | 0.669 (0.595~0.742) | 0.711 (0.643~0.779) | 0.576 (0.546~0.605) |
| Lactate | 0.786 (0.760~0.812) | 0.722 (0.688~0.755) | 0.654 (0.605~0.703) | 0.606 (0.545~0.667) | 0.586 (0.504~0.668) | 0.539 (0.458~0.619) | 0.555 (0.484~0.625) | 0.574 (0.509~0.639) | 0.662 (0.633~0.69) |
| WBC | 0.713 (0.674~0.752) | 0.701 (0.665~0.737) | 0.683 (0.636~0.729) | 0.630 (0.556~0.703) | 0.615 (0.53~0.7) | 0.575 (0.488~0.662) | 0.489 (0.414~0.564) | 0.522 (0.453~0.591) | 0.644 (0.614~0.673) |
| TB | 0.614 (0.577~0.65) | 0.589 (0.545~0.633) | 0.596 (0.543~0.649) | 0.577 (0.516~0.638) | 0.506 (0.42~0.592) | 0.467 (0.373~0.56) | 0.414 (0.333~0.495) | 0.448 (0.384~0.511) | 0.572 (0.544~0.599) |
| PT | 0.681 (0.647~0.715) | 0.623 (0.585~0.66) | 0.616 (0.565~0.667) | 0.635 (0.579~0.691) | 0.633 (0.571~0.694) | 0.644 (0.573~0.715) | 0.648 (0.578~0.718) | 0.643 (0.584~0.702) | 0.634 (0.607~0.661) |
| Creatinine | 0.690 (0.655~0.725) | 0.640 (0.599~0.68) | 0.594 (0.539~0.649) | 0.5645 (0.509~0.62) | 0.561 (0.495~0.626) | 0.558 (0.489~0.627) | 0.506 (0.438~0.573) | 0.518 (0.452~0.584) | 0.615 (0.588~0.642) |

CI, confidence interval; TB, total bilirubin; PT, prothrombin time; WBC, white blood cell
