# Supplementary material for: Time-varying discrimination accuracy of longitudinal biomarkers for the prediction of mortality compared to assessment at fixed time point in severe burns patients
Source: BMC Emerg Med. 2021 Jan 6;21:1. doi: 10.1186/s12873-020-00394-z (PMC7786914; doi:10.1186/s12873-020-00394-z)
Supplement: Supplementary file 2 — Additional file 2: Supplementary Table 2. Time varying Performance of updated biomarker using ID approach (AUC with 95% CI). [file 12873_2020_394_MOESM2_ESM.docx]

Supplementary Table 2. Time varying Performance of updated biomarker using ID approach (AUC with 95% CI)

|  | week1 | week2 | week3 | week4 | week5 | week6 | week7 | week8 | c-index |
| --- | --- | --- | --- | --- | --- | --- | --- | --- | --- |
| Platelet | 0.938 (0.922~0.954) | 0.956 (0.946~0.966) | 0.958 (0.941~0.975) | 0.970 (0.956~0.984) | 0.974 (0.96~0.988) | 0.980 (0.968~0.992) | 0.980 (0.968~0.992) | 0.987 (0.973~1) | 0.930 (0.919~0.941) |
| Lactate | 0.801 (0.771~0.83) | 0.775 (0.739~0.81) | 0.810 (0.774~0.846) | 0.853 (0.809~0.897) | 0.868 (0.82~0.916) | 0.901 (0.854~0.948) | 0.909 (0.858~0.96) | 0.922 (0.873~0.97) | 0.786 (0.758~0.814) |
| WBC | 0.653 (0.616~0.69) | 0.707 (0.675~0.739) | 0.753 (0.709~0.796) | 0.813 (0.763~0.862) | 0.817 (0.748~0.886) | 0.770 (0.691~0.849) | 0.714 (0.618~0.809) | 0.703 (0.609~0.797) | 0.684 (0.658~0.711) |
| TB | 0.745 (0.713~0.777) | 0.820 (0.795~0.845) | 0.910 (0.888~0.932) | 0.937 (0.912~0.962) | 0.900 (0.849~0.950) | 0.897 (0.841~0.952) | 0.910 (0.858~0.961) | 0.946 (0.908~0.983) | 0.782 (0.757~0.807) |
| PT | 0.872 (0.850~0.894) | 0.871 (0.848~0.894) | 0.905 (0.881~0.929) | 0.933 (0.908~0.957) | 0.900 (0.846~0.953) | 0.897 (0.834~0.96) | 0.898 (0.835~0.961) | 0.950 (0.917~0.982) | 0.862 (0.843~0.881) |
| Creatinie | 0.873 (0.850~0.895) | 0.876 (0.854~0.897) | 0.838 (0.805~0.871) | 0.813 (0.761~0.864) | 0.733 (0.653~0.813) | 0.726 (0.634~0.818) | 0.666 (0.568~0.764) | 0.715 (0.626~0.803) | 0.828 (0.809~0.848) |

CI, confidence interval; TB, total bilirubin; PT, prothrombin time; WBC, white blood cell
