# Supplementary material for: Time-varying discrimination accuracy of longitudinal biomarkers for the prediction of mortality compared to assessment at fixed time point in severe burns patients
Source: BMC Emerg Med. 2021 Jan 6;21:1. doi: 10.1186/s12873-020-00394-z (PMC7786914; doi:10.1186/s12873-020-00394-z)
Supplement: Supplementary file 3 — Additional file 3: Supplementary Table 3. Time varying Performance of baseline biomarkers using CD approach (AUC with 95% CI). [file 12873_2020_394_MOESM3_ESM.docx]

Supplementary Table 3. Time varying Performance of baseline biomarkers using CD approach (AUC with 95% CI)

|  | week1 | week2 | week3 | week4 | week5 | week6 | week7 | week8 |
| --- | --- | --- | --- | --- | --- | --- | --- | --- |
| Platelet | 0.562 (0.504~0.620) | 0.570 (0.496~0.643) | 0.611 (0.531~0.691) | 0.622 (0.507~0.737) | 0.514 (0.351~0.677) | 0.605 (0.401~0.808) | 0.857 (0.793~0.920) | 0.888 (0.829~0.946) |
| Lactate | 0.756 (0.714~0.798) | 0.710 (0.649~0.77) | 0.623 (0.555~0.691) | 0.600 (0.486~0.713) | 0.592 (0.412~0.772) | 0.469 (0.289~0.649) | 0.409 (0.223~0.594) | 0.550 (0.356~0.743) |
| WBC | 0.689 (0.632~0.745) | 0.679 (0.608~0.749) | 0.612 (0.519~0.705) | 0.618 (0.506~0.73) | 0.603 (0.435~0.77) | 0.511 (0.29~0.731) | 0.427 (0.284~0.570) | 0.263 (0.073~0.452) |
| TB | 0.617 (0.567~0.667) | 0.599 (0.523~0.674) | 0.654 (0.572~0.736) | 0.612 (0.513~0.711) | 0.421 (0.23~0.612) | 0.483 (0.285~0.68) | 0.512 (0.375~0.649) | 0.489 (0.303~0.674) |
| PT | 0.635 (0.583~0.687) | 0.596 (0.532~0.660) | 0.596 (0.515~0.676) | 0.659 (0.569~0.748) | 0.637 (0.484~0.789) | 0.564 (0.406~0.722) | 0.672 (0.577~0.767) | 0.674 (0.533~0.814) |
| Creatinie | 0.648 (0.597~0.699) | 0.633 (0.567~0.699) | 0.573 (0.493~0.653) | 0.574 (0.480~0.668) | 0.587 (0.428~0.745) | 0.540 (0.315~0.764) | 0.601 (0.443~0.758) | 0.276 (0.215~0.336) |

TB, total bilirubin; PT, prothrombin time; WBC, white blood cell
