# Supplementary material for: Time-varying discrimination accuracy of longitudinal biomarkers for the prediction of mortality compared to assessment at fixed time point in severe burns patients
Source: BMC Emerg Med. 2021 Jan 6;21:1. doi: 10.1186/s12873-020-00394-z (PMC7786914; doi:10.1186/s12873-020-00394-z)
Supplement: Supplementary file 4 — Additional file 4: Supplementary Table 4. Time varying Performance of updated biomarker using CD approach (AUC with 95% CI). [file 12873_2020_394_MOESM4_ESM.docx]

Supplementary Table 4. Time varying Performance of updated biomarker using CD approach (AUC with 95% CI)

|  | week1 | week2 | week3 | week4 | week5 | week6 | week7 | week8 |
| --- | --- | --- | --- | --- | --- | --- | --- | --- |
| Platelet | 0.871 (0.841~0.900) | 0.923 (0.899~0.946) | 0.944 (0.922~0.966) | 0.788 (0.694~0.881) | 0.989 (0.979~0.999) | 0.655 (0.445~0.864) | 0.953 (0.913~0.992) | 0.999 (0.997~1.000) |
| Lactate | 0.699 (0.657~0.741) | 0.756 (0.692~0.819) | 0.867 (0.83~0.904) | 0.729 (0.648~0.81) | 0.991 (0.983~0.999) | 0.787 (0.669~0.904) | 0.999 (0.998~1.000) | 0.938 (0.887~0.989) |
| WBC | 0.572 (0.517~0.627) | 0.615 (0.540~0.689) | 0.679 (0.585~0.773) | 0.695 (0.589~0.800) | 0.994 (0.989~0.999) | 0.897 (0.818~0.975) | 0.532 (0.228~0.836) | 0.659 (0.391~0.927) |
| TB | 0.595 (0.543~0.647) | 0.686 (0.628~0.743) | 0.822 (0.768~0.875) | 0.746 (0.664~0.827) | 0.982 (0.971~0.992) | 0.438 (0.113~0.763) | 0.935 (0.893~0.977) | 0.960 (0.926~0.994) |
| PT | 0.737 (0.693~0.781) | 0.719 (0.654~0.783) | 0.775 (0.719~0.83) | 0.730 (0.615~0.844) | 0.983 (0.973~0.992) | 0.566 (0.245~0.887) | 0.975 (0.952~0.998) | 0.841 (0.761~0.921) |
| Creatinie | 0.850 (0.815~0.885) | 0.811 (0.736~0.885) | 0.850 (0.792~0.908) | 0.599 (0.498~0.699) | 0.861 (0.827~0.895) | 0.288 (0.201~0.375) | 0.652 (0.337~0.967) | 0.525 (0.367~0.683) |

TB, total bilirubin; PT, prothrombin time; WBC, white blood cell
